# Advances in the Care of Primary Immunodeficiencies (PIDs): from Birth to Adulthood

**DOI:** 10.1007/s10875-017-0401-y

**Published:** 2017-05-18

**Authors:** Nizar Mahlaoui, Klaus Warnatz, Alison Jones, Sarita Workman, Andrew Cant

**Affiliations:** 1 French National Reference Center for Primary Immune Deficiencies (CEREDIH), Necker Enfants Malades University Hospital, Assistance Publique-Hôpitaux de Paris, Paris, France; 2 Pediatric Immuno-Haematology and Rheumatology Unit, Necker Enfants Malades University Hospital, Assistance Publique-Hôpitaux de Paris, Paris, France; 3 INSERM UMR 1163, Laboratory of Human Genetics of Infectious Diseases, Necker Branch, Paris, France; 4 Sorbonne Paris Cité, Imagine Institute, Paris Descartes University, Paris, France; 5 Center for Chronic Immunodeficiency (CCI), Medical Center—University of Freiburg, Faculty of Medicine, University of Freiburg, Freiburg, Germany; 6 Immunology Department, Great Ormond Street Hospital for Children NHS Foundation Trust, London, UK; 7 Department of Immunology, Royal Free London NHS Foundation Trust, 2nd Floor, Pond Street, Hampstead, London, NW3 2QG UK; 8 Great North Children’s Hospital, & Institute for Cellular Medicine University of Newcastle, Newcastle upon Tyne, NE4 1LP UK

**Keywords:** Primary immunodeficiency, diagnosis, management, reference centers/networks, transition care, transfer clinic, teenagers, young adults, continuity of care

## Abstract

Primary immunodeficiencies (PIDs) are a widely heterogeneous group of inherited defects of the immune system consisting of many clinical phenotypes with at least 300 underlying genetic deficits currently known. Patients with PIDs can present with, or develop during the course of their life, a susceptibility to recurrent and chronic infection along with autoimmune, allergic, inflammatory, and/or proliferative disorders, all potentially leading to end-organ damage. In recent years, a combination of basic and clinical research has greatly improved understanding of the underlying immunological and genetic defects in PIDs, leading to improved diagnosis, classification, and treatment approaches. In this review, we consider some of the key understandings that should direct diagnostic and treatment approaches in PID and offer insights into current and emerging management approaches and the lifelong care of patients from childhood through to adulthood.

## Introduction

The term “primary immunodeficiency (PID) diseases” describes a heterogeneous group of disorders which have in common poor or absent function in components of the innate and/or adaptive immune systems. New PIDs continue to be characterized as scientific and clinical understanding of these rare conditions advances [1, 2]. Recent decades have seen marked improvements in both the diagnosis and management of PIDs that have altered the outlook for many patients but which at the same time bring new challenges and add new complexities to the care of patients with PIDs. Since the early 1990s, molecular understanding of PIDs has increased greatly and there are now over 300 recognized and defined PIDs [1, 3]. In terms of the numbers of patients affected by PIDs, the European Society for Immunodeficiencies (ESID) Registry captures patient data from over 125 centers across Europe, and in 2014 recorded information on more than 20,000 PID patients [4]. The existence of such registries and collaboration between centers managing patients with PID helps to support better understanding of the epidemiological analyses of PID genotypes and phenotypes and is also crucial for the study and development of improved diagnostic and treatment interventions.

Although general awareness about PIDs remains patchy, there is much active and ongoing research. In this article, based on the proceedings of a symposium held during the 2015 International Primary Immunodeficiencies Congress (IPIC 2015), we consider contemporary approaches to the diagnosis of PIDs and how improvements in the knowledge of pathophysiology and in our ability to both diagnose and characterize PIDs have changed patient management in recent decades, leading to greater survival in children who used to have a shortened life expectancy. The natural history of disease course has been greatly modified by improvements in management and care. We consider the current and future therapeutic approaches and treatment opportunities that may bring further advances in patient care, and we reflect on how to support and manage the growing number of PID patients, particularly in their transition from pediatric to adult healthcare services.

### Diagnosis of PIDs—a Continually Evolving Story

Early diagnosis of PIDs remains a key goal; and in considering the available literature and evidence, it is important to draw a distinction between diagnostic techniques and innovations that have allowed for the discovery and description of “new” disorders, versus the diagnostic investigations that can be employed today in everyday clinical practice. Access to genetic tests in routine practice varies between specialist centers and countries. Today, the wait for results from many genetic tests can take months. Moving forward, what is needed are easily accessible, affordable, and rapid tests for all known PIDs.

Diagnostic discoveries and advances have considerably changed and improved the management of patients with PID in recent years [5]. Patients who 15–20 years ago would have defied categorization, or been without a clear diagnosis, might today benefit from novel diagnostic modalities, hope for a more definitive diagnosis, and therefore more appropriate management. Examples from our own case files (Box 1) as well as from the medical literature attest to how PID diagnosis has evolved and continues to evolve. In the case example of the first young female patient given in Box 1, until the papers of Lilic et al. at the turn of the millennium and the seminal publication by van de Veerdonk et al. in 2011 [6–9], it was not understood that gain-of-function STAT1 mutations impairing IL-17 immunity lay behind the deregulation of cytokines and the defective Th1 and Th17 responses seen in the PID of chronic mucocutaneous candidiasis.

**Table:** Box 1. Historical case examples: Case examples that took ~20 years before a definitive diagnosis was reached (Andrew Cant personal case file examples)

Girl—born 1987
• 3 months of age: candida (oral and napkin)
• Oesophageal strictures
1988/9
• 1 year: repeated LRTI; diarrhoea; FTT; bronchiectasis; CD4 lymphopenia No Igs; CMV in urine
• Chronic mucocutaneous candidiasis
Persistent and debilitating
Selective (*C. albicans*)
No systemic candidiasis
Autoimmunity (AIRE): multi-organ specific (polyendocrinopathy—APS-1) excluded
• Immunodeficiency: ? cause—selective to candida?
2011
• Diagnosis in 2011: STAT1 mutation
*LRTI* lower respiratory tract infection, *FTT* failure to thrive, *CMV* cytomegalovirus, *Igs* immunoglobulin, *AIRE* autoimmune regulator
Girl—born 1995
2001
• Ear and chest infections for 2–3 years
• CT scan—bronchial wall thickening
• PFTs normal
• Mother reported having ear & chest infections as child (IgG 4.5)
2007
• No naïve T or CSM B cells
• Cx glands settled
• AR hyper IgM excluded
“CVID” but IVIG Rx declined by parents
2014
• PI3KCD sequencing normal
• PI3KR1 G > A splice site mutation
• “APDS2” (mother same defect)
*PFT* pulmonary function test, *CSM* class-switched memory, *Cx* chest X-ray, *AR* autosomal recessive, *IgM/G* immunoglobulin M/G, *IVIG* intravenous immunoglobulin, *PI3KCD & PI3KR1* genes encoding the catalytic subunit of phosphatidylinositol 3-kinase δ (PI3K δ) and phosphoinositide-3-kinase, regulatory subunit 1, respectively, *APDS2* activated PI3K delta syndrome 2

Today, advances in our ability to diagnose certain PIDs much earlier in childhood have the potential to radically alter patient outcomes and survival [5] . One such example is the development of diagnostic modalities such as T cell receptors excision circle assays with the potential to detect severe combined immune deficiency (SCID) at birth from Guthrie dried blood spot samples—an advance that could allow for earlier and potentially curative intervention in a condition that, if undiagnosed, is associated with almost 100% mortality within the first 2 years of life. It has been clearly demonstrated that early diagnosis is associated with better outcomes in infants affected by SCID [10, 11]. There are therefore increasing calls for SCID diagnosis to become part of mandatory newborn disease screening [12].

Another example of change and continuing evolution in PID is our improved understanding and approach to the diagnosis of common variable immunodeficiency disorders (CVIDs) [13–15]. The term CVID describes a heterogeneous set of disorders that are collectively characterized by defective antibody production and which can have a highly variable clinical presentation typified by recurrent infection of the respiratory tract [16–18]. Readers are referred to recent publications for fuller details of the diagnostic criteria that apply in CVID [13–15], ESID Working Clinical Criteria (available here: www.esid.org/working-parties/Registry), and to recent publications describing current PID and CVID classifications [1].

While survival of patients with CVIDs has improved in recent decades due in part to the wider availability and advances in immunoglobulin replacement therapy, this has exposed a risk of morbidity and mortality in CVID due to secondary, non-infectious complications of disease that may include autoimmunity and inflammatory diseases, functional and structural lung disease, liver diseases, and the development of malignancies [15, 16]. We now appreciate that these secondary complications need to be actively searched out, as part of any diagnosis and patient phenotyping, as well as throughout continuing patient assessment [18, 19].

**Fig. 1:**
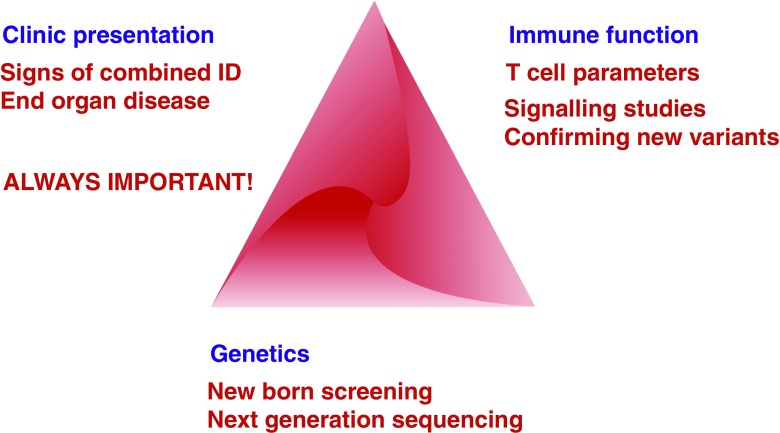
The three key diagnostic aspects of immunodeficiency to consider in CVID

Diagnosis on the basis of clinical presentation and close clinical scrutiny of the patient and their history remain of paramount and primary importance; but today, they are not sufficient alone to reach an accurate diagnosis of a CVID because of the large degree of overlap in phenotypes and the variable presentation of different genetic disorders, which may present like CVID but which do not constitute a CVID diagnosis [13, 14] (Fig. 1).

Among patients diagnosed with CVID, the number with undiagnosed combined immunodeficiency is generally underestimated, and this is a group that needs urgently to be identified through better clinical, laboratory, and increasingly genetic assessment.

The infection profile in CVIDs has been examined in several reports, such as those from the French DEF-I national study in which it was found that upper respiratory tract infections (URTIs) are highly prevalent and often manifest early as presenting features in CVID [20]. Unusual, opportunistic infections need to be interpreted as potential signs of a combined immunodeficiency [21], and patients presenting with these need to be further evaluated even when formally fulfilling the criteria for CVID. Similarly, clinical signs of immune dysregulation—often termed as complex CVID patients—should alert for a diagnostic work-up for combined immunodeficiency and end-organ disease(s).

As many as 30% of patients with CVID have autoimmune manifestations [16]. Based on next-generation sequencing, increasingly, combined immunodeficiencies are being discovered among CVID-like patients [ 22 ] . It is important to recognize and dissect out signs of immune dysregulation and autoimmunity—including inflammatory disorders—and be alert to the possibility of symptoms suggestive of underlying comorbidities (such as autoimmunity, lymphoproliferative disorders, or organ damage including cytopenia, granulomatous diseases, lymphoma, interstitial lung disease, bronchiectasis, splenomegaly, or autoimmune enteropathy) that all warrant further investigation for combined immunodeficiency by in-depth T cell analysis (*See* “Immune dysregulation—Pathogenic Concepts”).

Indeed, we recommend that any patient with a suspected diagnosis of so-called complex CVID should be referred to PID-specialist centers. Assessments of immune function should aim to delve beyond the primary hypogammaglobulinaemia that characterizes CVID [13], to include close analyses of serum IgG, IgA, and IgM levels and the assessment of specific antibody responses, together with thorough evaluation of the patient’s small lymphocyte panel and B (especially of switched memory B cells and CD21low B cells) and T cell parameters (especially of naïve CD4 T cells) using methods and cut-offs that exclude other diagnoses, as far as it is possible, and better define the immune profile of the patient with a CVID [15] (*See* ESID Working Clinical Criteria [23] and *See* below “Immune dysregulation—Pathogenic Concepts”).

While a genetic work-up is not recommended as a routine in the initial evaluation of all suspected CVID, exceptions should be made, such as in patients with a known consanguineous background or positive family history and in instances where laboratory studies strongly suggest an underlying genetic disease (*See* subsection below on emerging role for genetics). Genetic investigations are necessary in all patients with a complex CVID presentation and history in order to examine early on for monogenic defects presenting with immune dysregulation.

Next-generation sequencing (NGS) has almost superseded conventional Sanger DNA sequencing, and although not widely available in routine clinical practice, looks set to revolutionize genomic research and is already helping to identify new genetic syndromes with immune dysregulation and deficiency [5, 24–31] . NGS has revealed that the phenotype of single gene defects can vary more than expected and that many genetic defects have overlapping phenotypes. Genetic analyses using NGS have shown, for example, that mutations in lipopolysaccharide responsive beige-like anchor protein (LRBA) lead to an immune deficiency characterized by T and B cell defects, autophagy and apoptosis, and a clinical phenotype of childhood onset hypogammaglobulinaemia and autoimmunity [24]. NGS has identified loss of function mutations in protein kinase C delta as underlying a B cell hyperproliferation syndrome [25] and gain-of-function mutations in the PI3K-phosphoinositide 3-kinase (*PIK3CD*) gene in activated PI3kinase delta syndrome (APDS) patients [26, 27].

#### Immune Dysregulation—Pathogenic Concepts

Better understanding of the key pathomechanisms of immune dysregulation at play in CVIDs may hold the key to improved diagnosis and treatment of CVIDs and other PIDs in the future [22, 32].

Our concepts of the activation of the immune system are founded on the core principles that the immune system is designed around self/non-self-recognition, assessment of danger and integration of the signals between and inside participating cells [33, 34]. In conditions of immune dysregulation, it therefore follows that we might expect to find disturbed selection or production of immune cells, altered activation of immune cells and mediators, impaired regulatory T cell (Treg) homeostasis or function, and increased danger signalling [22].

In recent years, several defects in signalling molecules have demonstrated the association of disturbed antigen receptor signalling and autoimmune manifestations. Thus, patients with deficiency in the inducible T cell kinase (ITK) deficiency [35] or in the stromal interaction molecule 1 (STIM1) often present with clinical signs of disturbed immune tolerance. Protein kinase C delta (PKC-delta) deficiency is linked with defective B cell apoptosis and hyperproliferation and manifests as a systemic lupus erythematosus (SLE) phenotype [25, 36, 37]. In the APDS gain-of-function mutations of the phosphatidylinositol-3-OH kinase (PI3K), p110-delta or its regulatory component p85-alpha affects immune responses and stimulates abnormal cell proliferation, lymphadenopathies, and B cell lymphoma [26, 27].

The underlying cause and the clinical impact of reduced regulatory T cells seen in many CVID patients with autoimmunity are not understood but may contribute to the complexity of the immune dysregulation [38]. However, CTLA-4 deficiency has underlined how altered Treg function and immune regulation can lead to secondary immunodeficiency in several patients previously diagnosed as “CVID” [27, 28].

**Fig. 2:**
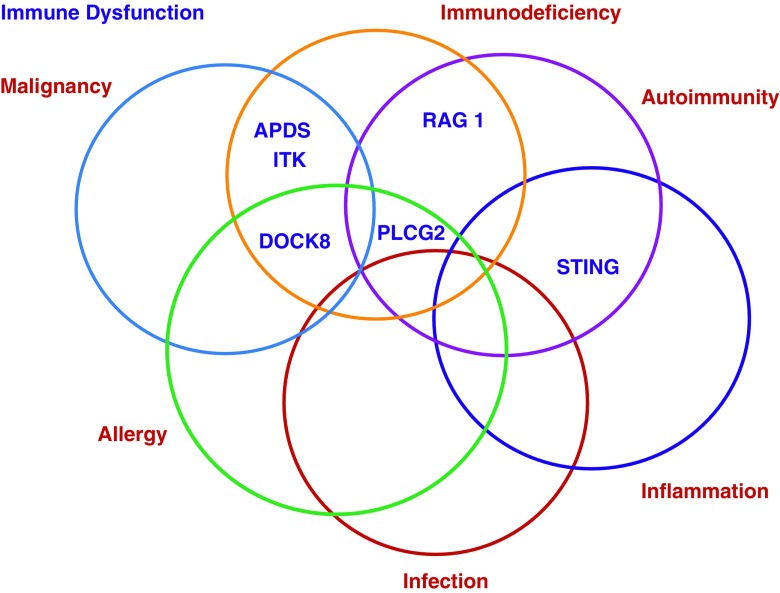
The overlap and interconnectivity of states in which immune dysfunction plays a part in disease pathology (image devised by A. Cant)

There has also been evidence for links between inflammation and the up-regulation of interferon (IFN)-responsive genes in patients with complex CVID [39]. An altered cross-talk between gut microbiota, the intestinal epithelium, and the immune system may lead to bacterial translocation, Toll-like receptor (TLR)-activation, and IFN-induction, and this appears to contribute to the CD4 T cell exhaustion and functional impairment observed in some CVID patients [40, 41]. Our current understanding of these mechanisms contributing to the pathogenesis of immune dysregulation is still very rudimentary (Fig. 2).

### Current and Future Perspectives on PID Management

Management decisions for patients with PIDs need to be based more and more on all three aspects of diagnosis—the clinical presentation, an understanding of the patient’s immune function test results, and the underlying genetic defect. Currently, in most cases, management decisions will be led by the clinical presentation.

This may involve the use of anti-infectious prophylaxis and the aggressive treatment of infections; however, for most patients with antibody deficiency, the cornerstone of therapy is immunoglobulin replacement therapy, the goal of which is to prevent most infections [18, 42–44]. Many studies show that immunoglobulin replacement therapy significantly reduces the rate of acute and chronic infection and greatly improves survival. Seminal studies that have helped guide and direct the optimal use of immunoglobulin replacement therapy in the management of CVID for example include the 22-year patient follow-up study of Lucas et al. and studies assessing the impact of immunoglobulin replacement therapy on pneumonia incidence in PIDs [44, 45]. These studies underscore the importance of maintaining adequate trough plasma levels of IgG in order to control infection and significantly reduce the sequelae of acute and/or recurrent infections in PIDs. However, this treatment fails to control most secondary autoimmune and inflammatory complications.

Therefore, an improved understanding of the immunopathological and genetic defects in PIDs is key and will help to shed light on potential new targets and the possible role of immune-modifying agents in the future management of PIDs, autoimmune diseases and other closely linked conditions involving immune dysregulation, malignancy, and inflammation. For example, in patients with PIDs where there are gain-of-function mutations affecting PI3K activity, such as APDS 1/2, there is emerging data from in vitro studies to suggest that hyperactivity of mTOR might be amenable to inhibition by mTOR inhibitors such as rapamycin and that this agent might impact on T cell defects in a manner that could theoretically, in turn, affect the clinical course of this PID [27]. Other avenues of research that may yield opportunities for therapeutic intervention include the study of various STAT mutations, such as STAT1 mutations associated with impairments in IL-17 immunity, and linked with chronic mucocutaneous candidiasis, and STAT3 gain-of-function mutations that are linked with altered T cell and cytokine signalling and with lymphoproliferation and autoimmunity with prominent cytopenias. Such pathologies suggest that there may be opportunities to study new molecules with pharmacological profiles such as IL-6 inhibition and JAK inhibition that could impact on STAT gain-of-function or decrease JAK and STAT3 phosphorylation and act to counter the underlying deficits that define certain PIDs [6–9, 46, 47].

Combined immunodeficiencies are not sufficiently treated by antibody replacement and some patients may require hematopoietic stem cell transplantation (HSCT). The role for HSCT continues to evolve for this subgroup of PIDs, as survival with cure of severe PIDs is now possible for up to 90% of patients undergoing HSCT for severe PIDs [48, 49]. With newborn screening, SCID is a condition detectable at birth before secondary complications develop; and with such screening, early HSCT offers a curative option [12]. Allogeneic HSCT may also offer an option for some patients with complex CVID, although evidence to date suggests that this therapeutic approach should be considered in carefully selected patients ideally at a time when only little or no organ damage or chronic infection has occurred [50]. It is clear that advances in donor matching and new hematopoietic stem cell (HSC) manipulation techniques, such as HLA-haploidentical HSCT associated with alpha-beta+ T and B cell depletion, are already changing our approach to HSCT in PIDs [51], and such advances together with reductions in graft versus host disease (GVHD) incidence, improved options for reduced intensity conditioning and better anti-infectious treatment options, will combine to ensure better outcomes following HSCT in PID patients.

In parallel with improvements in approaches and practices relating to HSCT, active clinical studies involving gene therapy have been pioneered in the field of PIDs [3]. Lessons from HSCT have allowed for the study of ex vivo gene transfer in hematopoietic stem cells as a means of effecting phenotype correction. First clinical trials performed with gamma retroviral vectors for adenosine deaminase severe combined immunodeficiency (ADA-SCID), X-linked SCID (SCID-X1), and Wiskott-Aldrich syndrome (WAS) identified that gene therapy is a valid therapeutic option in patients without an HLA-identical donor. To date, no insertional mutagenesis events have been reported in ADA-SCID cases managed with gene therapy, although insertional oncogenesis has been reported in SCID-X1 and WAS, prompting the development of vector constructs based on self-inactivating retroviral or lentiviral vectors [3, 52, 53].

Thus a number of challenges still remain, with many PID patients still facing protracted times to diagnosis and requiring lifelong chronic treatment rather than having curative options. Nevertheless, the long-term outcomes of PID patients have improved greatly in recent decades, such that many patients now survive longer and require new support paradigms as they age and mature.

### PID—Increasing Numbers of Patients Transitioning to Adult Care

Many PIDs emerge in childhood; and with improved therapeutic options, life expectancy for patients with PIDs has increased in recent years [13, 42]. This means that many more PID patients now transition from pediatric to adult healthcare services, leading to a greater emphasis on the importance of the transition process. Transition has been defined as “A purposeful, planned process that addresses the medical, psychosocial, and educational/vocational needs of adolescents and young adults with chronic physical and medical conditions as they move from child-centered to adult-oriented health care systems” [54].

Transition is an important step for young people with complex health needs or disabilities, and their families, and needs to be carefully planned and handled [55]. Some countries already have well-established transition services for patients with specific lifelong conditions that emerge in childhood, such as cystic fibrosis, diabetes mellitus, chronic kidney disease, and epilepsy; but, to date, transition pathways for PID have been less clearly defined. Of note, the International Patient Organisation for Primary Immunodeficiencies—IPOPI has devised a booklet on transition [56].

In the UK, Great Ormond Street Hospital (GOSH) is a national center involved in the care of pediatric patients with PIDs. Many of the children cared for at GOSH move on to the Royal Free London NHS Foundation Trust (RFH) in London for ongoing care in adulthood. The two centers have worked closely to establish best practice guidelines and integrated care pathways for all adolescents with PID requiring transition to the RFH (as well as other adult centers in the UK).

The ongoing needs of children with PID depend on their underlying condition and previous management. Broadly, they fall into two main groups:

(i) Those who have lifelong conditions that require long-term medical treatment. Most of these are patients with various forms of primary antibody deficiency who require lifelong immunoglobulin replacement therapy, and many of these are on home immunoglobulin treatment.
(ii) Increasing numbers of patients who have undergone HSCT or gene therapy for severe PID. All of these individuals require long-term monitoring. Some are fully immune reconstituted and lead normal healthy lives, requiring only an annual check-up and monitoring blood tests. However, significant numbers in this group have ongoing medical and/or psychological issues, and some remain on long-term Ig replacement therapy.

Several issues are particularly important to appreciate inpatients with a PID diagnosis approaching transition. Many of these children will have had their diagnosis since infancy, and they and their families will have forged close ties with pediatric care providers. Children and parents often develop very close relationships with the pediatric care team and the prospect of transferring to an adult environment, and losing the security of a familiar team can be daunting. Coupled with this, the stresses of change and maturation during adolescence, with issues of developing autonomy and self-reliance can impact on behaviors and attitudes. In addition, many children with a PID diagnosis have complex multi-system disorders. Some have conditions that have non-immunological manifestations, and some have complications of PID, and these individuals require multi-disciplinary/multi-specialty care into adulthood. Transition planning and transition pathways therefore need to take into account the multiple aspects of maturation along with the social and personal changes that will affect the diagnosis, treatment, treatment compliance, and autonomy of the future adult with PID. Appropriate guidance and support during transition and regarding different options for treatment and care are essential to promote and sustain good and acceptable compliance with long-term management plans.

**Fig. 3:**
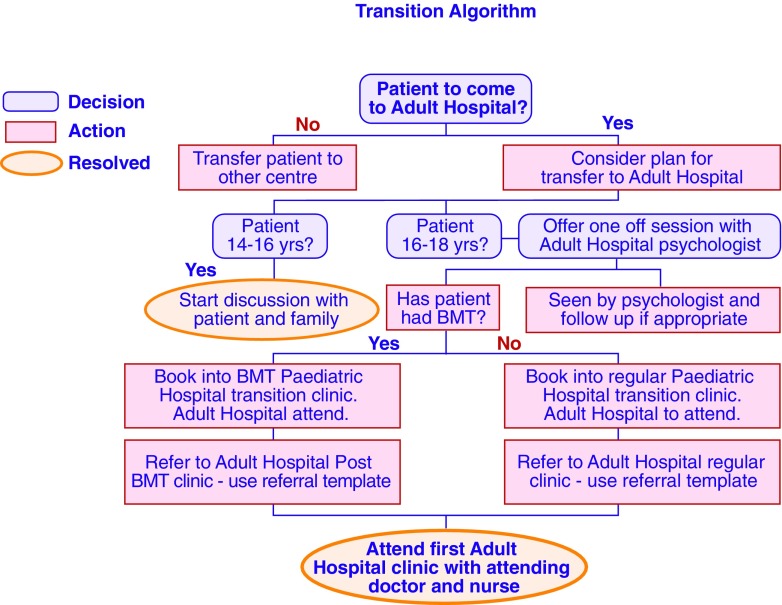
An example of a transition algorithm (courtesy of S. Workman)

At GOSH and the RFH, preparation for transition starts early, and discussions regarding impending change and the need for transition to adult services often begin with patients and families from the age of 12 years. The pediatric and adult centers run joint clinics and follow an agreed handover process (Fig. 3) designed to ensure that patients and their families enjoy a smooth transition, with no gaps in care, and have access to members of both the pediatric team and the adult team during the period of transition. The period of transition does not have a time limit: each patient will be assessed on an individual basis. When the patients have been successfully transferred, members of the GOSH team continue to have joint clinics at the RFH to maintain continuity of care. This structured process aims to ensure that patients and their families are confident of continuing, uninterrupted care and supports sharing of case data and experiences across the healthcare teams responsible for pediatric care, adolescent, and adult care.

## Conclusions

Primary immune deficiencies are a heterogeneous group of diseases defined by defects in the immune systems leading to increased susceptibility to infection, autoimmunity, immune dysregulation, inflammation, end-organ damage, and malignancy. Great strides have been made in the understanding of the pathogenesis and underlying immunological and genetic defects that define different PIDs and impact on prognosis and outcomes. Armed with this greater understanding of PIDs, opportunities for earlier recognition and diagnosis of otherwise life-threatening conditions have been made possible; and today, many patients with PIDs can be offered treatments tailored to their condition, which have the potential to improve outlook and survival. Mainstay treatments such as IVIG replacement therapy continue to be important for long-term patient care; and as more is understood about the causes of PIDs, opportunities for wider use of potentially curative options, such as HSCT and gene therapy, and for study and development of disease-targeted therapies improve. Patients with PIDs continue to need close assessment and care at specialist centers, where active research and study will pave the way for improved future and lifelong patient care.
